# Supplementary material for: Biofilm microenvironment triggered self-enhancing photodynamic immunomodulatory microneedle for diabetic wound therapy
Source: Nat Commun. 2023 Nov 23;14:7658. doi: 10.1038/s41467-023-43067-8 (PMC10667311; doi:10.1038/s41467-023-43067-8)
Supplement: Supplementary file 1 — Supplementary Information [file 41467_2023_43067_MOESM1_ESM.pdf]

Supplementary Information

Biofilm Microenvironment Triggered Self-Enhancing Photodynamic Immunomodulatory Microneedles for Diabetic Wound Therapy

Li Yang *et al.*

Supplementary Experimental Details

Materials

Ascorbic acid (Vc, >99%), dopamine hydrochloride (DA, >98%), LA (>99%), MB >94%, and selenite pentahydrate ($Na_2SeO_3 \cdot 5H_2O$, >98%) were purchased from Sigma-Aldrich. Chlorin e6 (Ce6, >94%) and 5,5-dimethyl-1-pyrroline N-oxide (DMPO, >97%) were obtained from Macklin. GSH and GSSG assay kit, NO assay kit and ROS assay kit (DCF-DA) were purchased from Beyotime. A microreduced GSH assay kit was obtained from Solarbio. Mouse IL-10 enzyme-linked immunoassay (ELISA) kit, mouse IL-1 ELISA kit, mouse IL-4 ELISA kit (IL-4), and mouse TNF-α ELISA kit were purchased from Meimian. Anti-mouse F4/80 Antibody, anti-mouse CD197 (CCR7) Antibody and anti-mouse CD206 (MMR) Antibody were obtained from Biolegend. Active nitrogen detection kit and live/dead bacterial staining kit were purchased from BBoxiProbe®. Hydroxyphenyl fluorescein was obtained from AAT Bioquest. Singlet oxygen sensor green was purchased from Thermo Fisher Scientific.

Examining anti-biofilm contribution of $^1O_2$, RNS and •OH by superposition

To examine the anti-biofilm contribution of each RS, the following four groups were set up:

(1) Control + 8 mM GSH group: No RS was generated after irradiation;

(2) C@P + 8 mM GSH group: only $^1O_2$ was generated after irradiation;

(3) C@PA + 8 mM GSH group: $^1O_2$ and RNS were generated after irradiation;

(4) SeC@PA + 8 mM GSH group: $^1O_2$, RNS and •OH were generated after irradiation.

Considering the scavenging effect of GSH on RS, we adjusted the concentration of nanoparticles in each group until the fluorescence intensity of $^1O_2$ in C@P + 8 mM GSH group, C@PA + 8 mM GSH group and SeC@PA + 8 mM GSH group showed no obvious difference, the fluorescence intensity of RNS in C@PA + 8 mM GSH group and SeC@PA + 8 mM GSH group showed no obvious difference. Subsequently, the anti-biofilm assay was performed with the adjusted concentrations of each group.

ELISA

To quantify cytokines in biofilm-infected diabetic wound, fresh wound tissues were added to PBS and ground into tissue homogenate. The supernatants were collected and then used to quantify the

protein concentration for each specimen by BCA protein assay kit (Beyotime). Concentration of different cytokines (IL-1, IL-4, IL-10 and TNF-α) was assessed by ELISA kits (MEIMIAN, China) and the absorbance was measured at 450 nm by a microplate reader (Molecular devices, China).

Western blotting

Western blotting assay was utilized to analyze the expressions of STAT6, JAK1 and ERK1/2. In brief, RAW264.7 cells were seeded into 6-well plates at the 1×10^6 cells per well. After adhesion, RAW264.7 cells were treated with C@PA (+), Se@PA and SeC@PA(+) for 6 h. Then, the cells were washed with PBS three times and added into lysis buffer on the ice. Afterwards, western blot test was performed to investigate the relative expression of STAT6, p-STAT6, JAK-1, p-JAK1, ERK1/2 and p-ERK1/2 in the above cell lysates.

Quantitative real-time PCR

RAW264.7 cells were treated with C@PA(+), Se@PA and SeC@PA(+) for 24 h. Total RNA was extracted with FineProtect Universal RNA Kit (GENFINE Biotech, China). A Nano Dro spectra-photometer (Nanodrop2000, Thermo Fisher Scientific, USA) was used to quantify the total RNA. The complementary DNA (cDNA) was further synthesized by using PrimeScript™ RT reagent Kit (Takara). The expression levels of genes were detected by using the synergy brands (SYBR) Green Select Master Mix (Bio-Rad) in triplicate on an ABI QuantStudio 5 cycler (Thermo Fisher). All mRNA expression levels were normalized to the *GAPDH* reference gene. The relative mRNA expression level of target gene was normalized to control sample and calculated using the $2^{-\Delta\Delta Ct}$ method. The forward and reverse primer sequences were designed and listed in Supplementary Table 2.

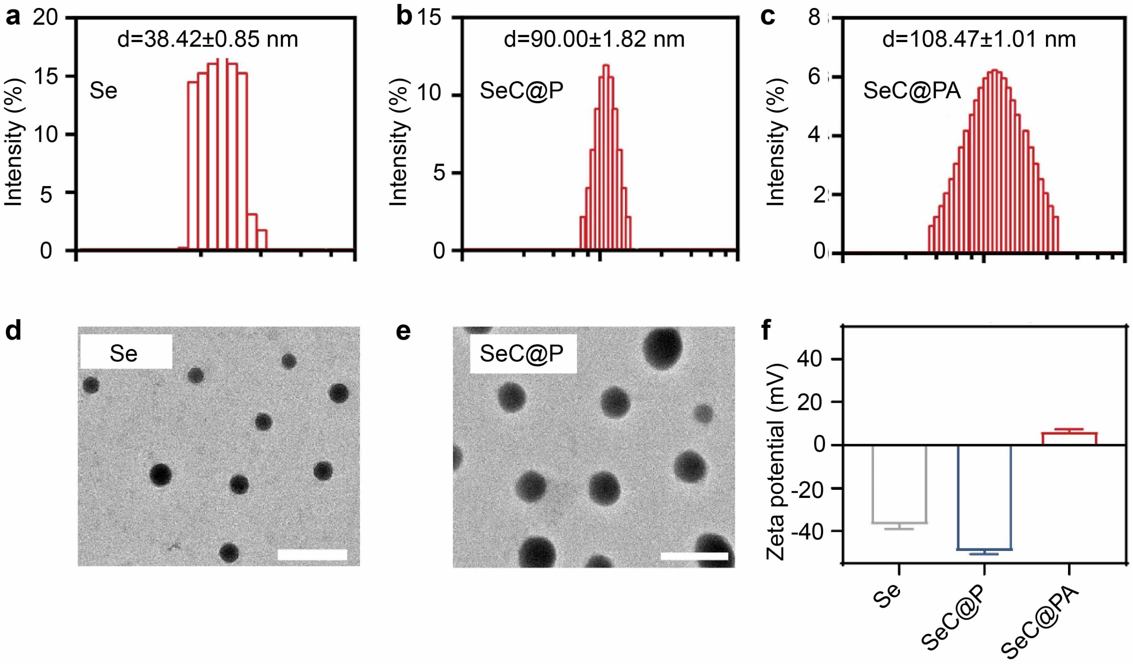

Supplementary Fig. 1. Nanoparticle characterization. a-c Hydrodynamic sizes of Se, SeC@P and SeC@PA measured by dynamic light scattering (DLS) (*n*=3 independent samples, mean ± SD). **d, e** Transmission electron microscopy (TEM) images of Se and SeC@P, respectively. Scale bar is 200 nm. Two independent experiments were performed and representative results are shown. **f** Zeta potential values of the nanoparticles (*n* = 3 independent samples, mean ± SD). Se: Se nanoparticles; SeC@P: Se-Ce6-PDA nanoparticles; SeC@PA: Se-Ce6-PDA-LA nanoparticles. Source data are provided as a Source Data file.

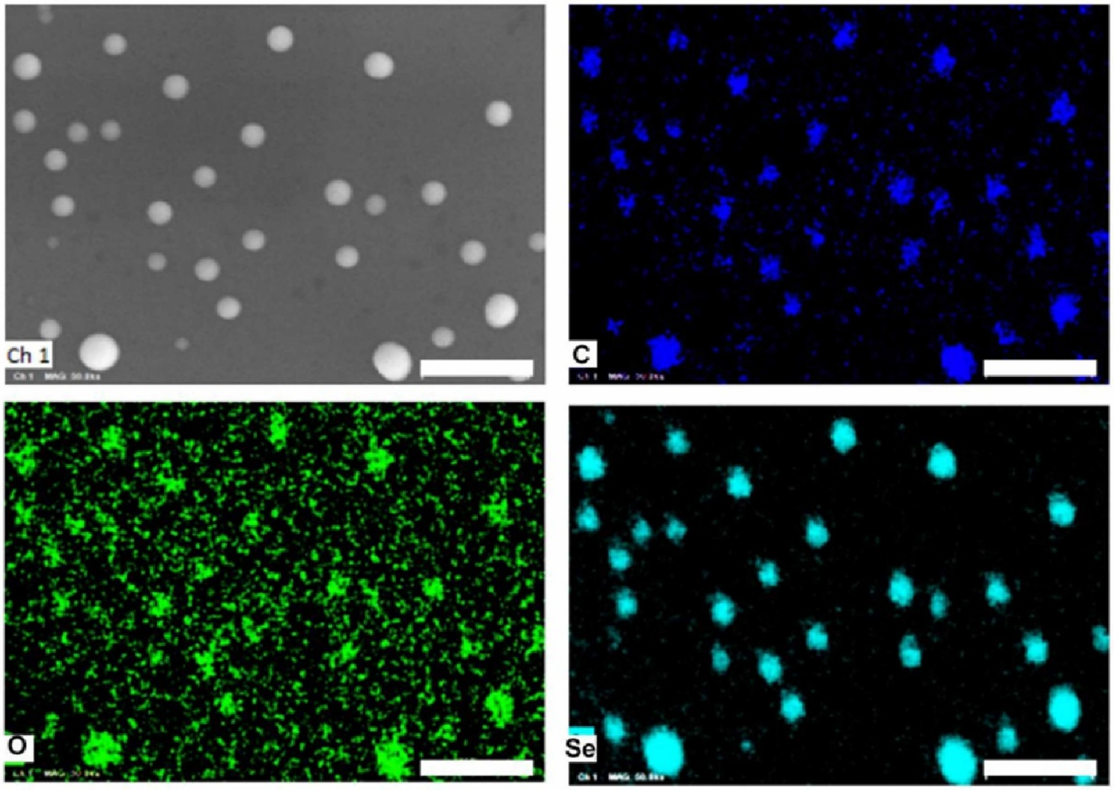

Supplementary Fig. 2. SEM image of SeC@PA in the condition of the EDS mapping analysis and corresponding EDS mapping results. Scale bar is 500 nm. Three independent experiments were performed and representative results are shown.

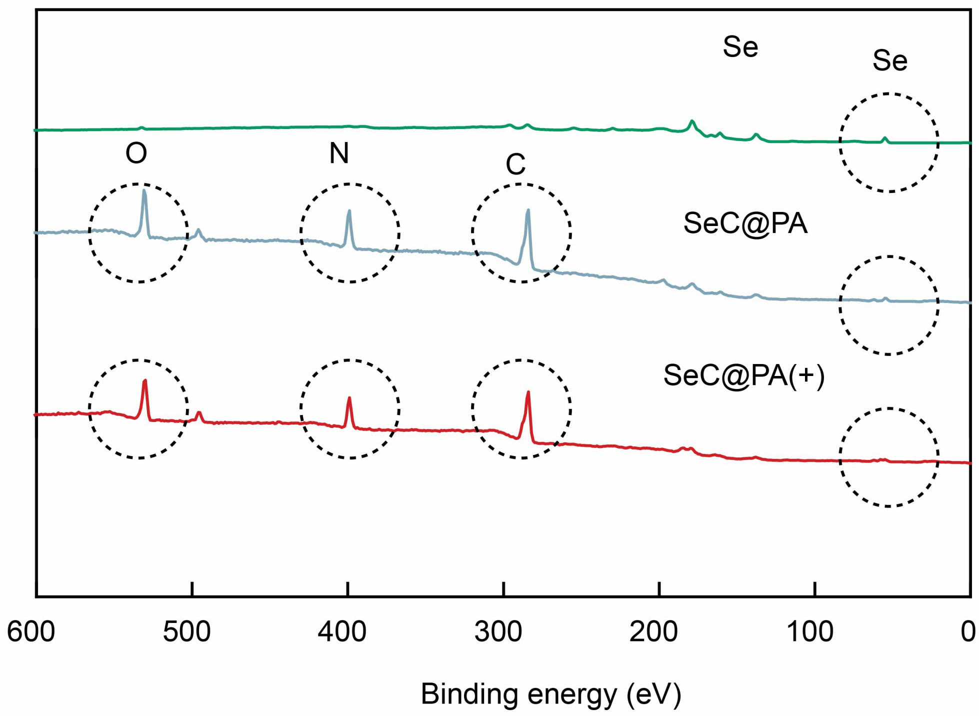

Supplementary Fig. 3. XPS spectra of Se, SeC@PA and SeC@PA(+). Se: Se nanoparticles; SeC@PA: Se-Ce6-PDA-LA nanoparticles; SeC@PA(+): Se-Ce6-PDA-LA nanoparticles under 660 nm irradiation (200 mW/cm^2) for 3 min. Source data are provided as a Source Data file.

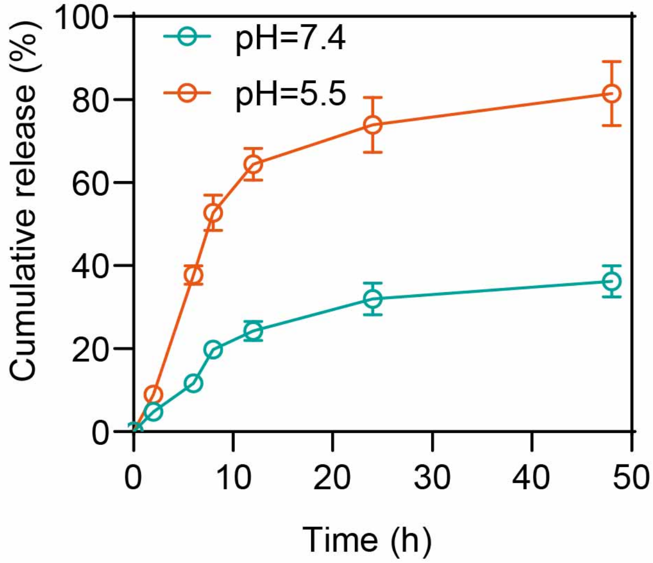

Supplementary Fig. 4. Ce6 release profile in PBS with different pH values (*n* = 3 independent samples, mean ± SD). Source data are provided as a Source Data file.

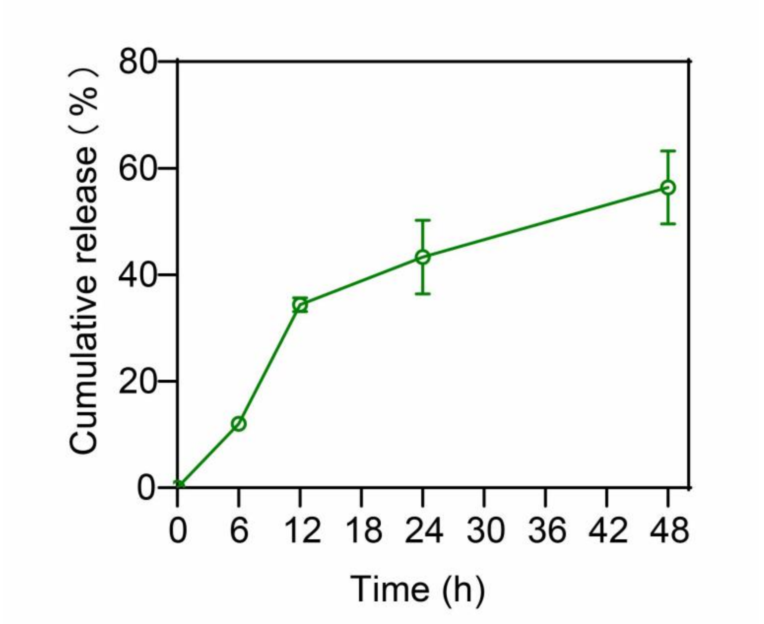

Supplementary Fig. 5. Se release profile in PBS at pH = 5.5 (*n* = 3 independent samples, mean ± SD). Source data are provided as a Source Data file.

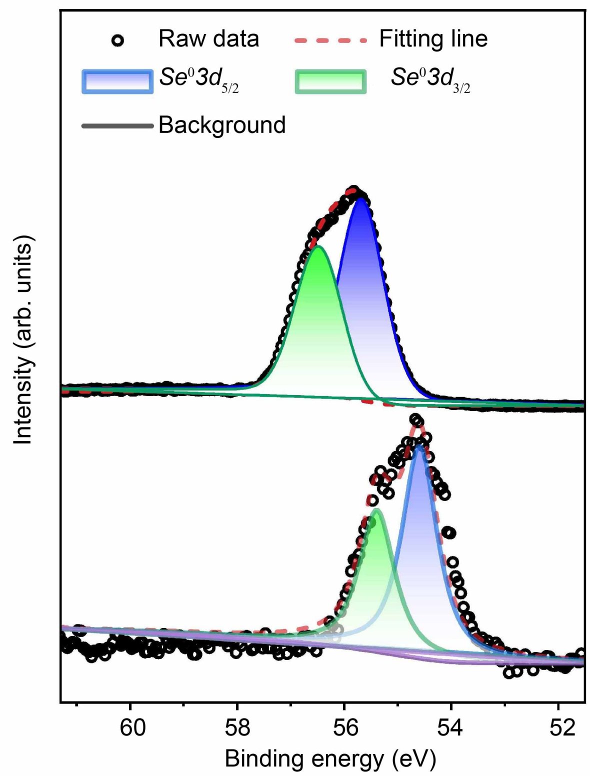

Supplementary Fig. 6. High-resolution *Se 3d* XPS spectra of Se and SeC@PA. Source data are provided as a Source Data file.

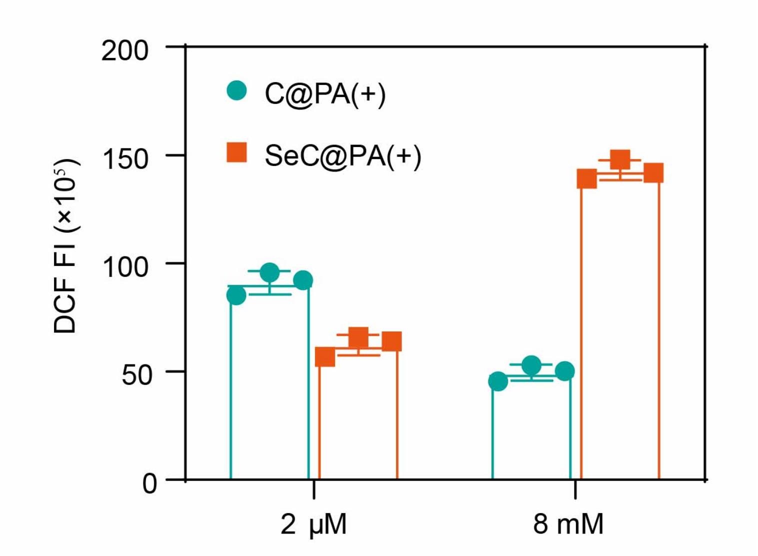

Supplementary Fig. 7. DCF fluorescence intensity of C@PA(+) and SeC@PA(+) under different GSH concentration (n = 3 independent samples; mean ± SD). Statistical significance was analyzed via two-tailed Student's t-test. Source data are provided as a Source Data file.

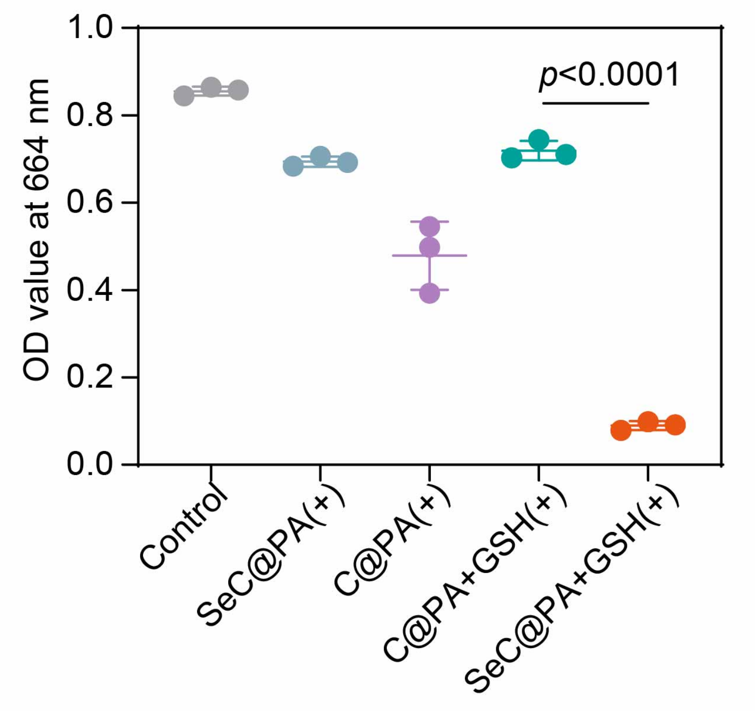

Supplementary Fig. 8. Absorbance values of methylene blue at 664 nm after different treatments (n = 3 independent samples; mean ± SD). Statistical significance was analyzed via one-way ANOVA with a Tukey post-hoc test. Source data are provided as a Source Data file.

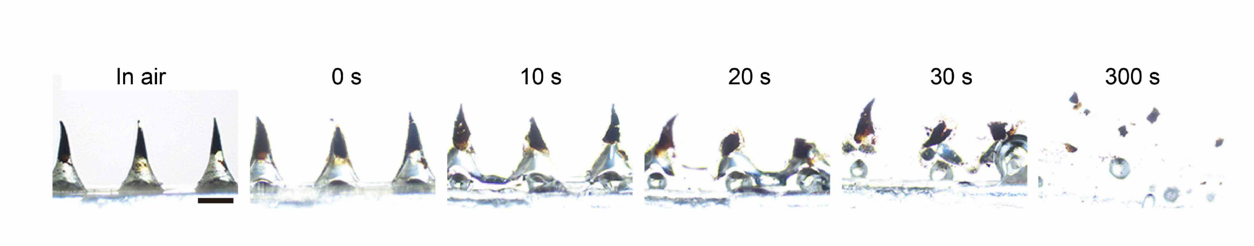

Supplementary Fig. 9. In vitro separation behavior of SeC@PA MN patch at different times in PBS. Scale bar is 500 μm. Two independent experiments were performed and representative results are shown.

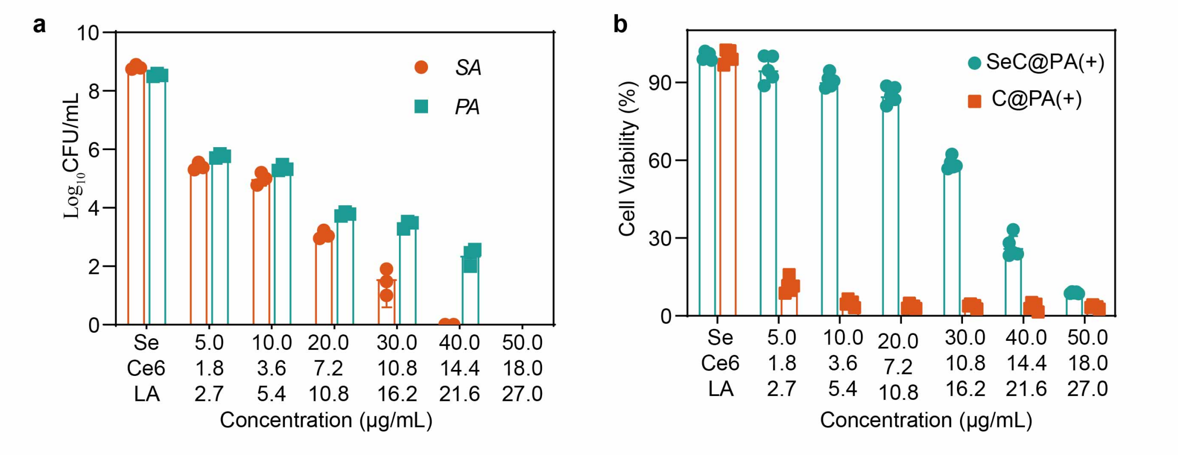

Supplementary Fig. 10. Bactericidal and cell viability results. a Bactericidal results of SeC@PA(+) with different concentrations characterized by the standard plate counting assay (n = 3 biologically independent samples; mean ± SD). **b** Cell viability of SeC@PA(+) and C@PA(+) with different concentration (n = 5 biologically independent samples; mean ± SD). Source data are provided as a Source Data file.

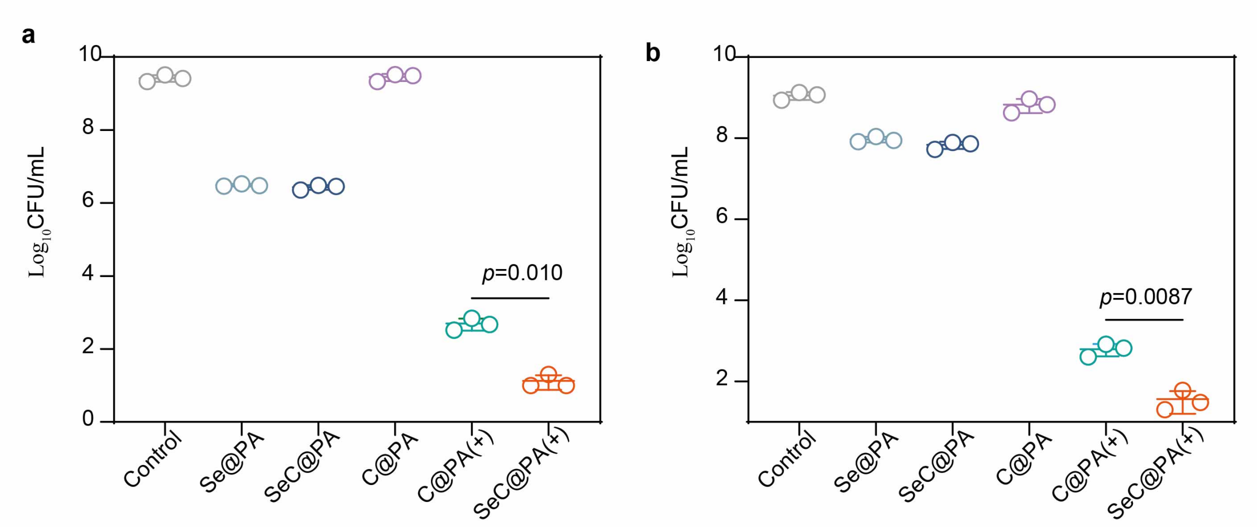

Supplementary Fig. 11. Bactericidal results. a Bactericidal results of different groups characterized by the standard plate counting assay in SA suspension (n = 3 biologically independent samples; mean ± SD). **b** Bactericidal results of different groups characterized by the standard plate counting assay in PA suspension (n = 3 biologically independent samples; mean ± SD). Statistical significance was analyzed via one-way ANOVA with a Tukey post-hoc test. Source data are provided as a Source Data file. Se@PA: Se-PDA-LA nanoparticles; SeC@PA: Se-Ce6-PDA-LA nanoparticles; C@PA: Ce6-PDA-LA nanoparticles; C@PA(+): Ce6-PDA-LA nanoparticles under 660 nm irradiation (200 mW/cm^2) for 3 min; SeC@PA(+): Se-Ce6-PDA-LA nanoparticles under 660 nm irradiation (200 mW/cm^2) for 3 min.

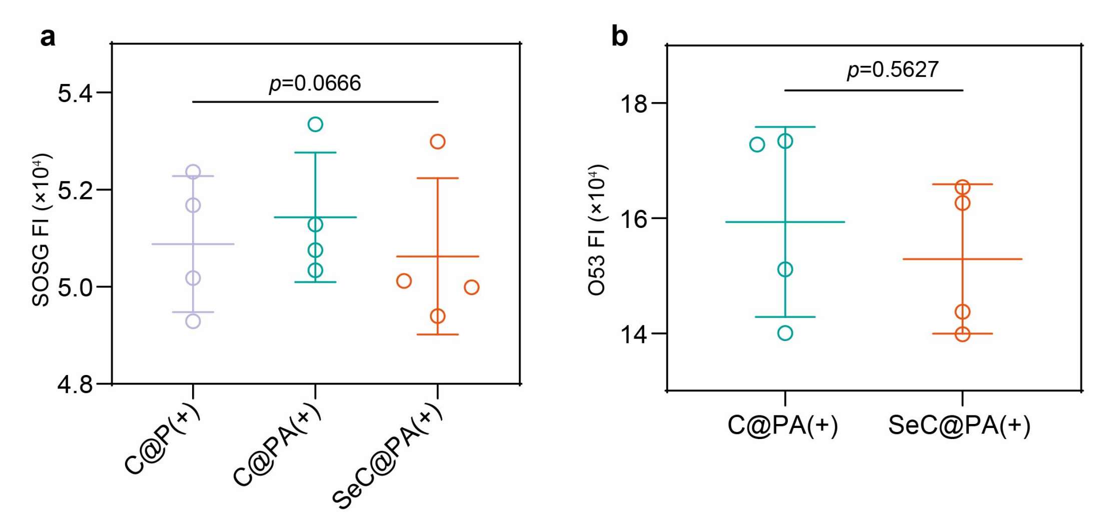

Supplementary Fig. 12. Fluorescence intensity. a Fluorescence intensity of SOSG and **b** O53 in different groups (n = 4 independent samples, mean ± SD). Statistical significance was analyzed via one-way ANOVA with a Tukey post-hoc test. Source data are provided as a Source Data file. C@P(+): Ce6-PDA nanoparticles under 660 nm irradiation (200 mW/cm^2) for 3 min; C@PA(+): Ce6-PDA-LA nanoparticles under 660 nm irradiation (200 mW/cm^2) for 3 min; SeC@PA(+): Se-Ce6-PDA-LA nanoparticles under 660 nm irradiation (200 mW/cm^2) for 3 min.

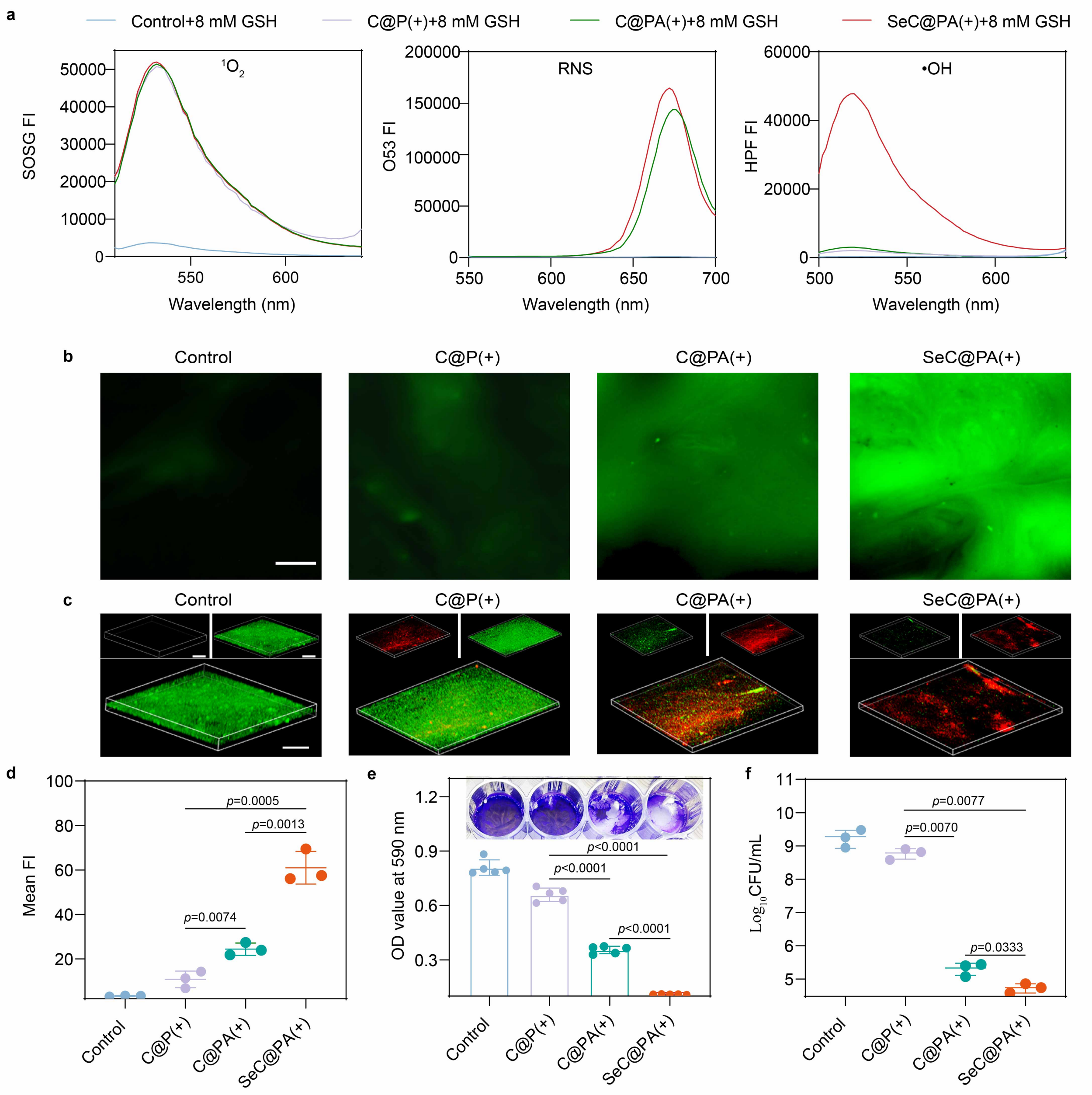

Supplementary Fig. 13. Anti-biofilm effect of different RS. **a** Fluorescence intensity of SOSG ($^1O_2$), O53 (RNS) and HPF (•OH) in different groups. **b** Detection of RS in the SA biofilm incubated with different nanoparticles after 660 nm irradiation (200 mW/cm^2) for 3 min. Scale bar is 500 μm. Three independent experiments were performed and representative results are shown. **c** Live/dead stain of SA biofilms with different treatments under 660 nm irradiation (200 mW/cm^2) for 3 min. Scale bar is 100 μm. Three independent experiments were performed and representative results are shown. **d** Semiquantitative statistics of the mean fluorescence intensity of DCF in SA biofilms treated with different groups (n = 3 biologically independent samples; mean ± SD). **e**

Representative images of crystal violet staining and absorbance values at 590 nm after treatment with different nanoparticles (*n* = 5 biologically independent samples; mean ± SD). **f** Bactericidal results of different nanoparticles characterized by the standard plate counting assay (*n* = 3 biologically independent samples; mean ± SD). Statistical significance was analyzed via one-way ANOVA with a Tukey post-hoc test. Source data are provided as a Source Data file. C@P(+): Ce6-PDA nanoparticles under 660 nm irradiation (200 mW/cm^2) for 3 min; C@PA(+): Ce6-PDA-LA nanoparticles under 660 nm irradiation (200 mW/cm^2) for 3 min; SeC@PA(+): Se-Ce6-PDA-LA nanoparticles under 660 nm irradiation (200 mW/cm^2) for 3 min.

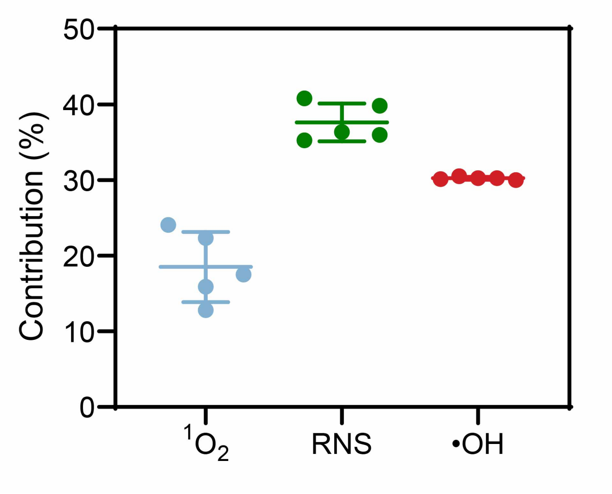

Supplementary Fig. 14. Anti-biofilm contribution of $^1O_2$, RNS and •OH (n = 5 biologically independent samples; mean ± SD). Source data are provided as a Source Data file.

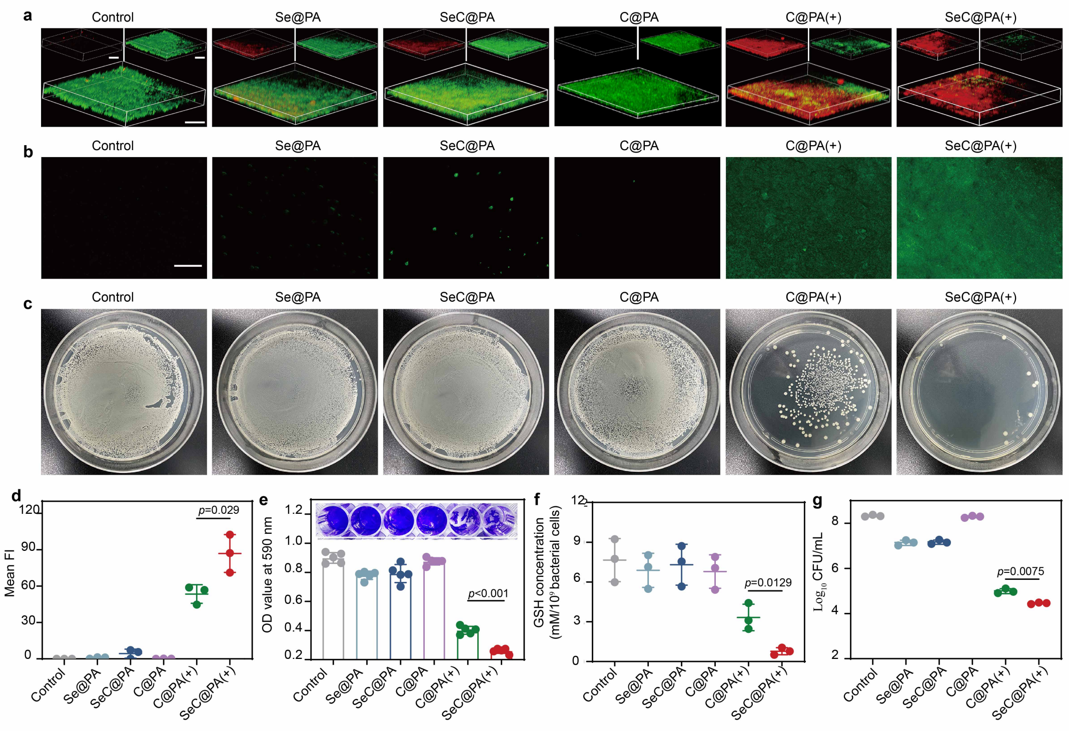

Supplementary Fig. 15. Anti-biofilm effect of nanoparticles. **a** Live/dead stain of PA biofilms with different treatments under 660 nm irradiation (200 mW/cm^2) for 3 min. Scale bar is 100 μm. Three independent experiments were performed and representative results are shown. **b** Detection of RS in the PA biofilm incubated with different nanoparticles after 660 nm irradiation (200 mW/cm^2) for 3 min. Scale bar is 500 μm. Three independent experiments were performed and representative results are shown. **c** Representative optical images of colony-forming units for PA biofilm suspension after different treatments. **d** Semiquantitative statistics of the mean fluorescence intensity of DCF in PA biofilms treated with different groups (n = 3 biologically independent samples; mean ± SD). **e** Representative images of crystal violet staining and absorbance values at 590 nm after treatment with different nanoparticles (n = 5 biologically independent samples; mean ± SD). **f** GSH concentration in PA biofilms after treatments with different nanoparticles (n = 3 biologically independent samples; mean ± SD). **g** Bactericidal results of different nanocarriers characterized by the standard plate counting assay (n = 3 biologically independent samples; mean ± SD). Statistical significance was analyzed via one-way ANOVA with a Tukey post-hoc test. Source data are provided as a Source Data file. C@PA: Ce6-PDA-LA nanoparticles; Se@PA: Se-PDA-LA nanoparticles; SeC@PA: Se-Ce6-PDA-LA nanoparticles; C@PA(+): Ce6-PDA-LA nanoparticles under 660 nm irradiation (200 mW/cm^2) for 3 min; SeC@PA(+): Se-Ce6-PDA-LA nanoparticles under 660 nm irradiation (200 mW/cm^2) for 3 min.

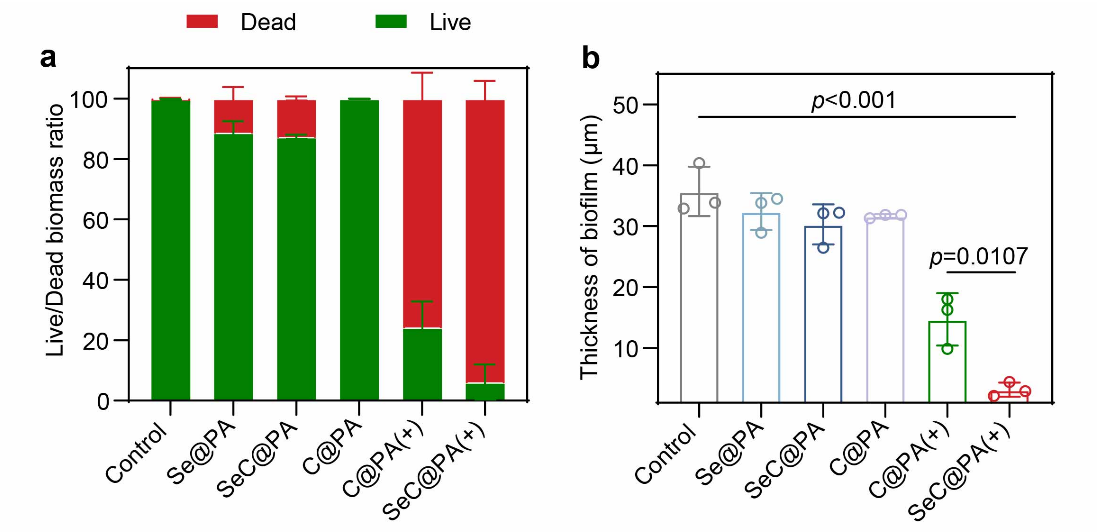

Supplementary Fig. 16. Live/dead biomass ratio and thickness of SA biofilm. a Live/dead biomass ratio of SA biofilm with different treatments (n = 3 biologically independent samples; mean ± SD). **b** Thickness of SA biofilm after different treatments (n = 3 biologically independent samples; mean ± SD). Statistical significance was analyzed via one-way ANOVA with a Tukey post-hoc test. Source data are provided as a Source Data file. C@PA: Ce6-PDA-LA nanoparticles; Se@PA: Se-PDA-LA nanoparticles; SeC@PA: Se-Ce6-PDA-LA nanoparticles; C@PA(+): Ce6-PDA-LA nanoparticles under 660 nm irradiation (200 mW/cm^2) for 3 min; SeC@PA(+): Se-Ce6-PDA-LA nanoparticles under 660 nm irradiation (200 mW/cm^2) for 3 min.

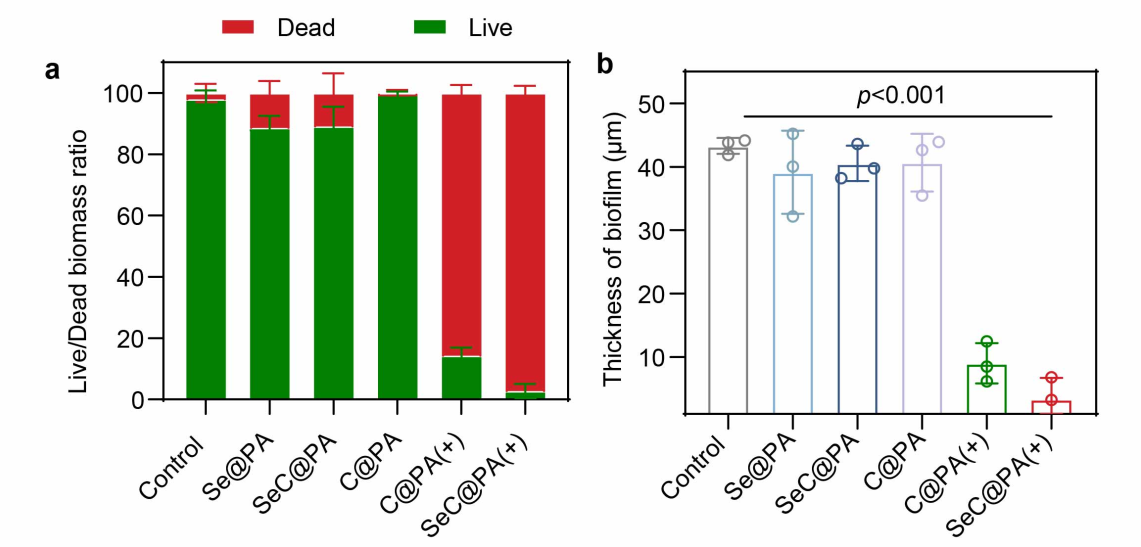

Supplementary Fig. 17. Live/dead biomass ratio and thickness of PA biofilm. a Live/dead biomass ratio of PA biofilm with different treatments (n = 3 biologically independent samples; mean ± SD). **b** Thickness of PA biofilm after different treatments (n = 3 biologically independent samples; mean ± SD). Statistical significance was analyzed via one-way ANOVA. Source data are provided as a Source Data file. C@PA: Ce6-PDA-LA nanoparticles; Se@PA: Se-PDA-LA nanoparticles; SeC@PA: Se-Ce6-PDA-LA nanoparticles; C@PA(+): Ce6-PDA-LA nanoparticles under 660 nm irradiation (200 mW/cm^2) for 3 min; SeC@PA(+): Se-Ce6-PDA-LA nanoparticles under 660 nm irradiation (200 mW/cm^2) for 3 min.

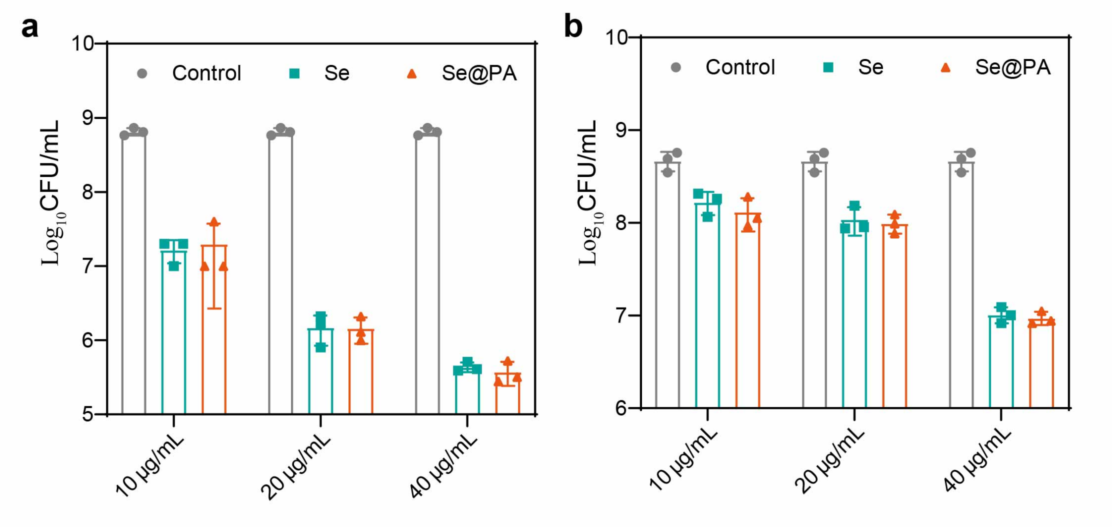

Supplementary Fig. 18. Bactericidal results. a Bactericidal results of Se and Se@PA with different concentrations in SA suspension characterized by the standard plate counting assay (n = 3 biologically independent samples; mean ± SD). **b** Bactericidal results of Se and Se@PA with different concentrations in PA suspension characterized by the standard plate counting assay (n = 3 biologically independent samples; mean ± SD). Source data are provided as a Source Data file. Se: Se nanoparticles; Se@PA: Se-PDA-LA nanoparticles.

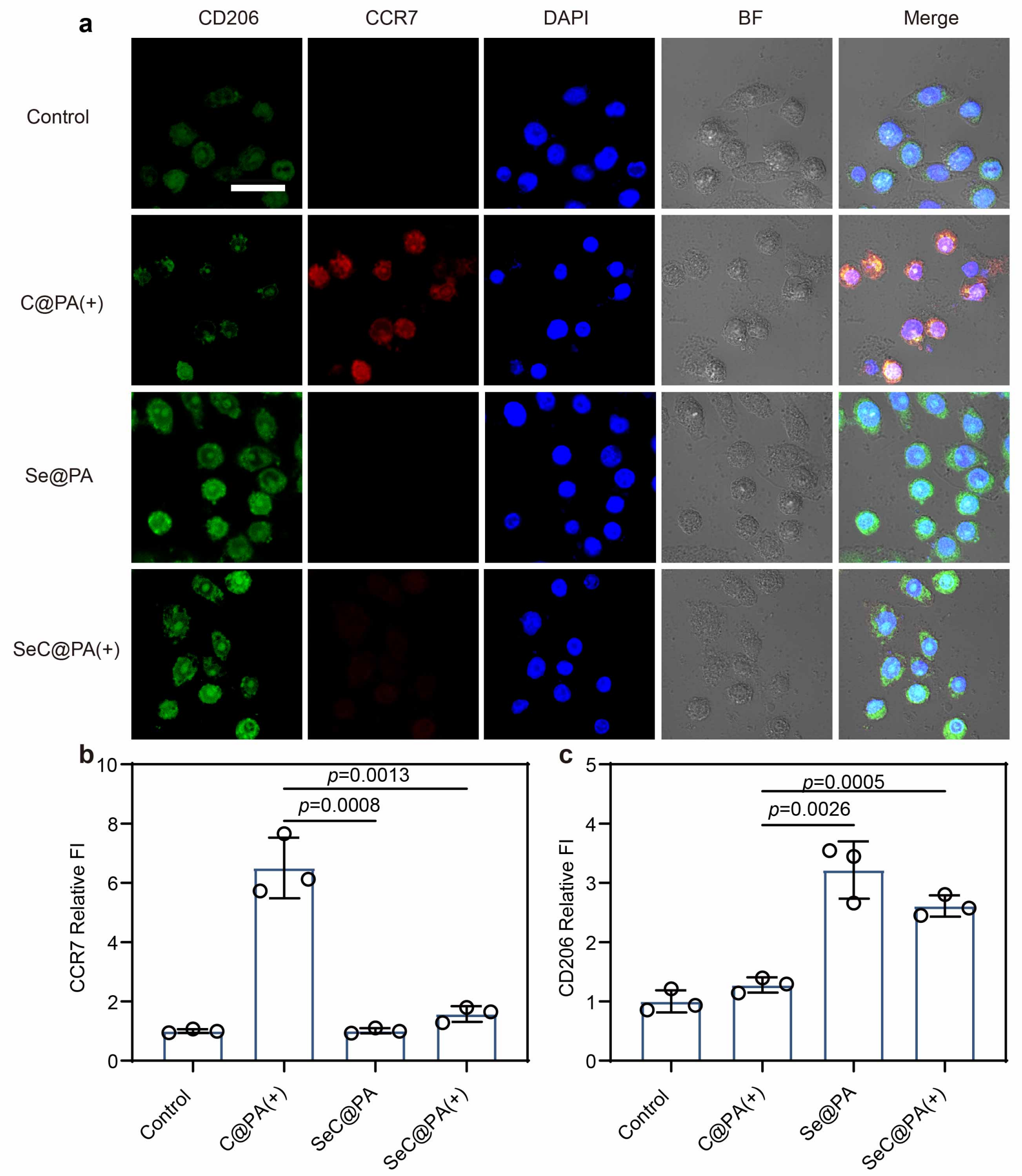

Supplementary Fig. 19. Staining images and semiquantitative statistics. a Representative images of CCR7 and CD206 staining of RAW264.7 cells after different treatments. Scale bar is 200 μm. Three independent experiments were performed and representative results are shown. **b, c** Semiquantitative statistics of the relative fluorescence intensity of CCR7 and CD206, respectively (n = 3 biologically independent samples; mean ± SD). Statistical significance was analyzed via one-way ANOVA with a Tukey post-hoc test. Source data are provided as a Source Data file. Se@PA: Se-PDA-LA nanoparticles; C@PA(+): Ce6-PDA-LA nanoparticles under 660 nm irradiation (200 mW/cm^2) for 3 min; SeC@PA(+): Se-Ce6-PDA-LA nanoparticles under 660 nm irradiation (200 mW/cm^2) for 3 min.

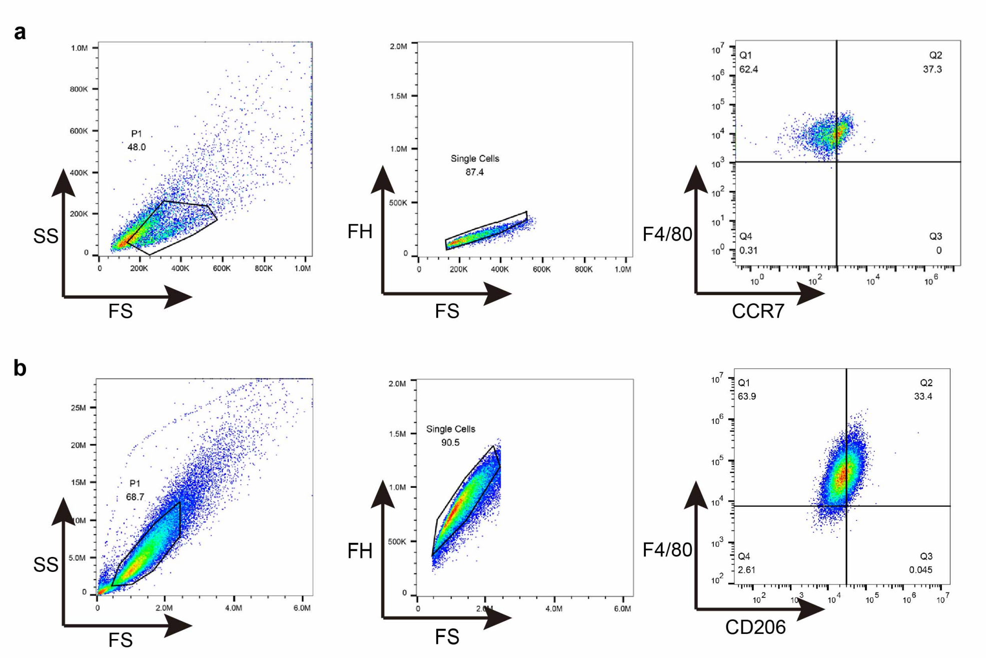

Supplementary Fig. 20. Gating strategy for identifying macrophages. a Gating strategy for identifying M1-type macrophages on Fig. 5a. **b** Gating strategy for identifying M2-type macrophages on Fig. 5a.

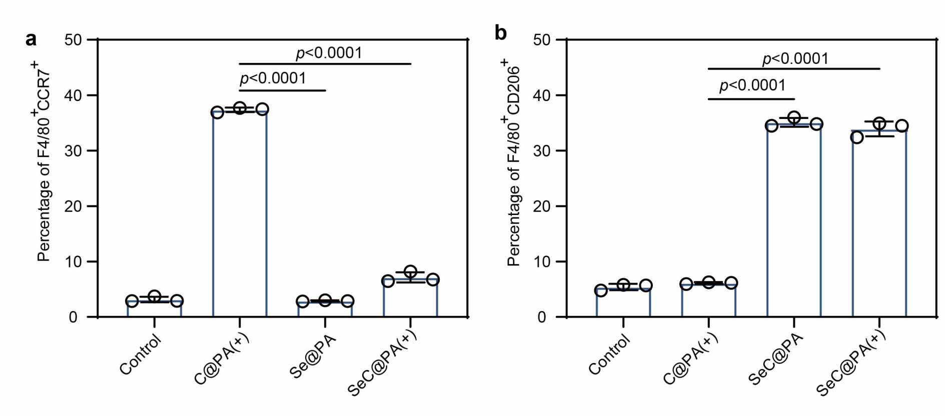

Supplementary Fig. 21. Percentages of macrophages. **a** Percentage of M1-type ($F4/80^{+}CCR7^{+}$) macrophages (n = 3 biologically independent samples, mean ± SD). **b** Percentage of M2-type ($F4/80^{+}CD206^{+}$) macrophages (n = 3 biologically independent samples, mean ± SD). Statistical significance was analyzed via one-way ANOVA with a Tukey post-hoc test. Source data are provided as a Source Data file. Se@PA: Se-PDA-LA nanoparticles; C@PA(+): Ce6-PDA-LA nanoparticles under 660 nm irradiation (200 mW/cm^2) for 3 min; SeC@PA(+): Se-Ce6-PDA-LA nanoparticles under 660 nm irradiation (200 mW/cm^2) for 3 min.

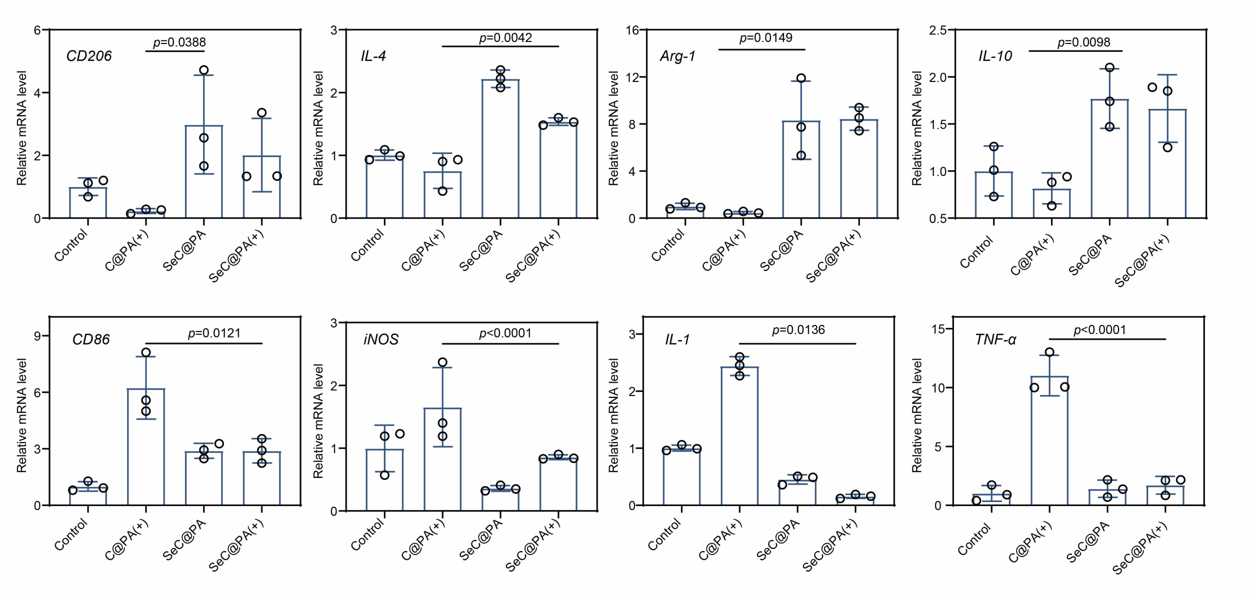

Supplementary Fig. 22. Relative mRNA expression of *CD206*, *Arg-1*, *IL-4*, *IL-10*, *CD86*, *iNOS*, *IL-1* and *TNF-α* in RAW264.7 with different treatments (n = 3 biologically independent samples, mean ± SD). Statistical significance was analyzed via one-way ANOVA with a Tukey post-hoc test. Source data are provided as a Source Data file. Se@PA: Se-PDA-LA nanoparticles; C@PA(+): Ce6-PDA-LA nanoparticles under 660 nm irradiation (200 mW/cm^2) for 3 min; SeC@PA(+): Se-Ce6-PDA-LA nanoparticles under 660 nm irradiation (200 mW/cm^2) for 3 min.

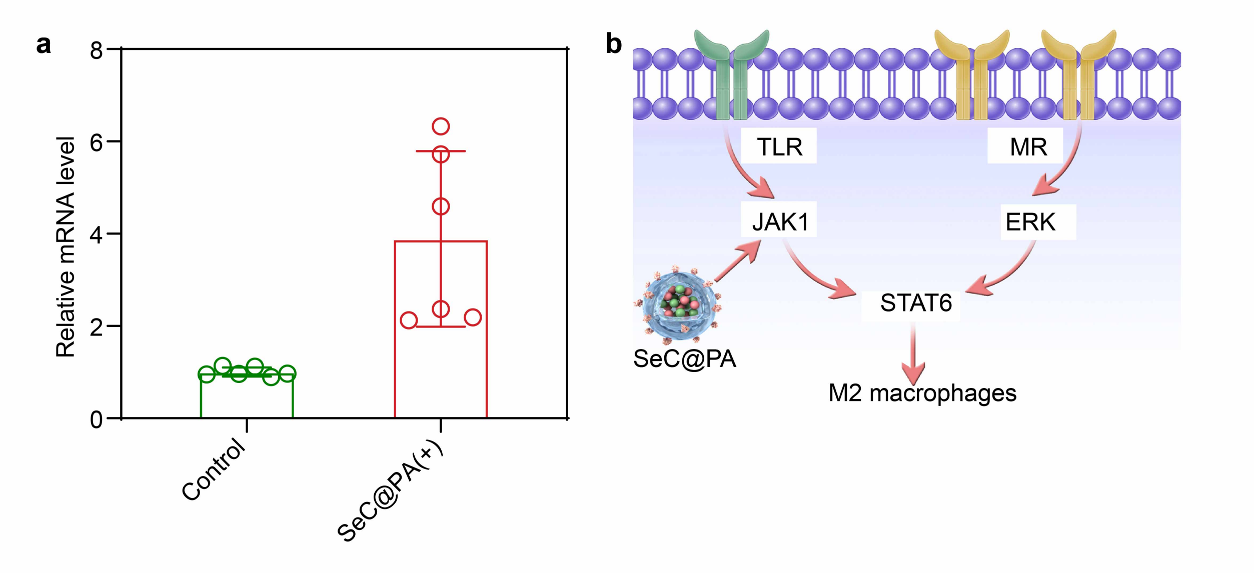

Supplementary Fig. 23. Relative mRNA expression and mechanism. a Relative mRNA expression of *STAT6* (n = 6 biologically independent samples; mean ± SD). **b** Mechanism of M2-type macrophage polarization induced by SeC@PA. Source data are provided as a Source Data file.

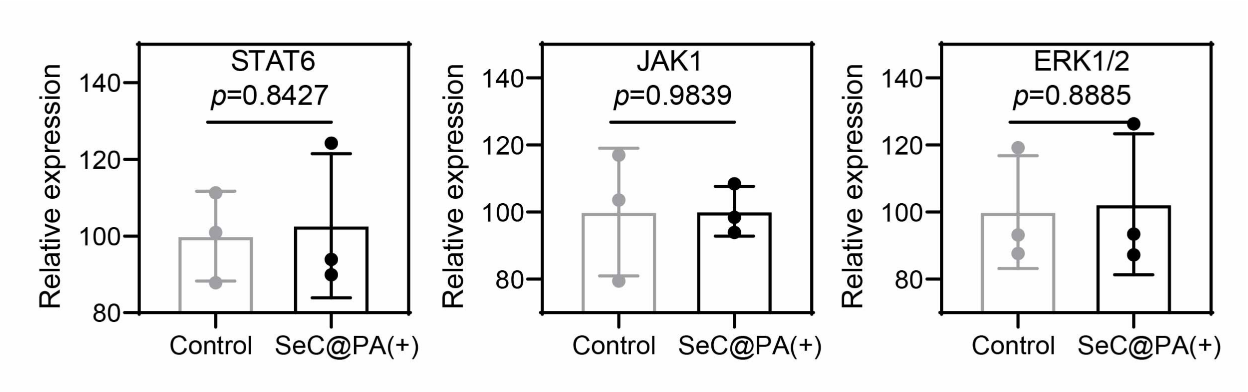

Supplementary Fig. 24. Expression levels of STAT6, JAK1 and ERK1/2 in the RAW264.7 cells determined by western blotting after treatments (n = 3 biologically independent samples; mean ± SD). Statistical significance was analyzed via two-tailed Student's t-test. Source data are provided as a Source Data file.

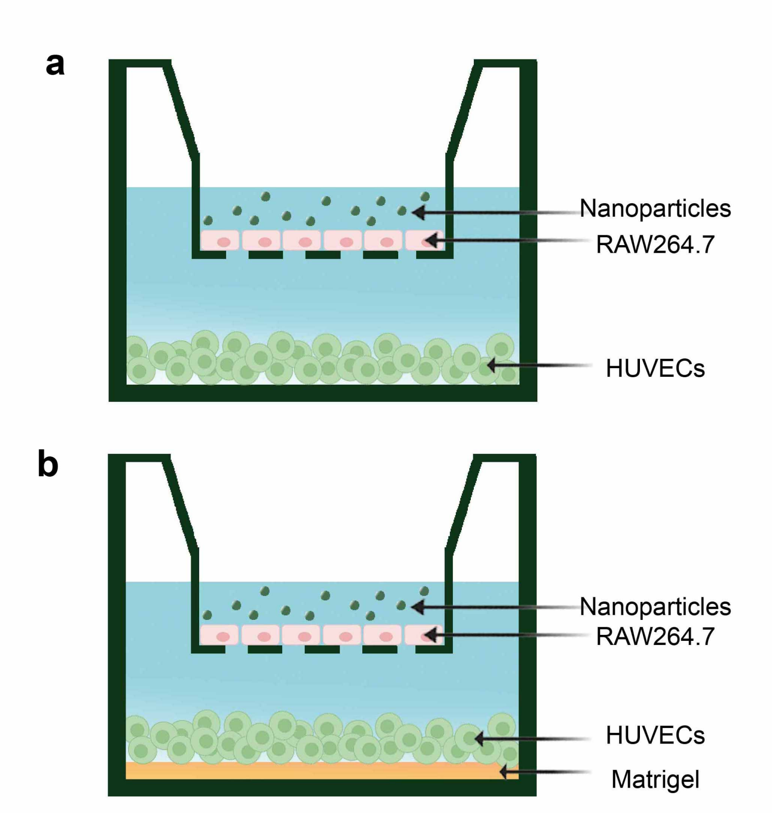

Supplementary Fig. 25. HUVECs. **a** Schematic representation for migration assay of HUVECs. **b** Schematic diagram for the tube formation assay of HUVECs.

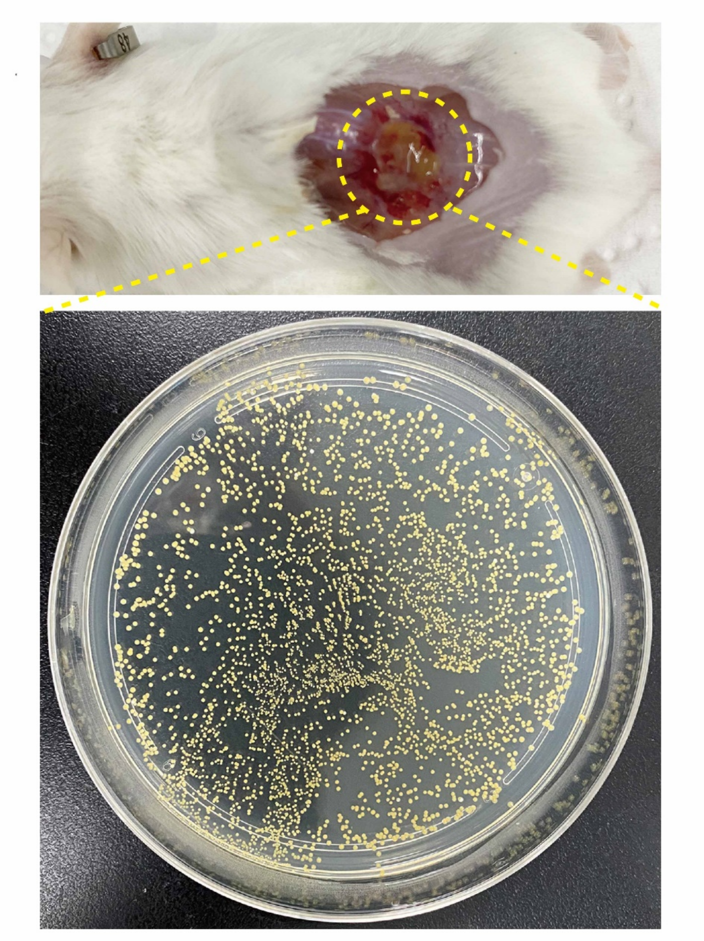

Supplementary Fig. 26. Photograph of biofilm-infected tissue and representative photograph of bacterial culture from the skin tissues of diabetic mouse wounds infected with biofilms.

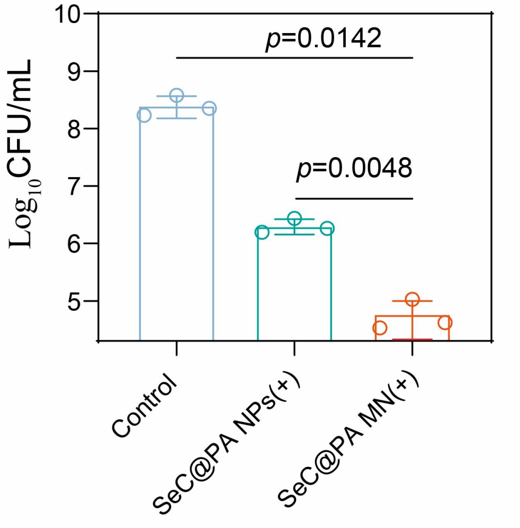

Supplementary Fig. 27. Bactericidal results at day 4 characterized by the standard plate counting assay (n = 3 biologically independent samples; mean ± SD). Statistical significance was analyzed via one-way ANOVA with a Tukey post-hoc test. Source data are provided as a Source Data file.

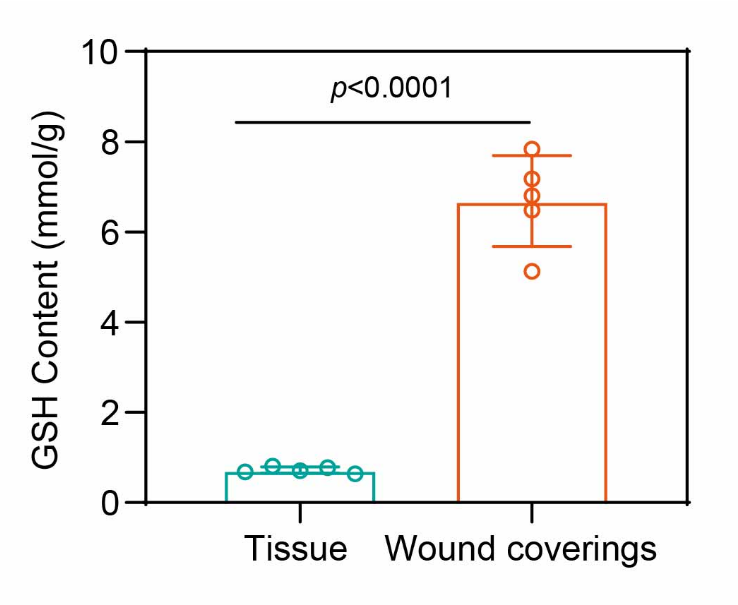

Supplementary Fig. 28. GSH content of tissue and wound coverings (n = 5 biologically independent samples; mean ± SD). Statistical significance was analyzed via two-tailed Student's t-test. Source data are provided as a Source Data file.

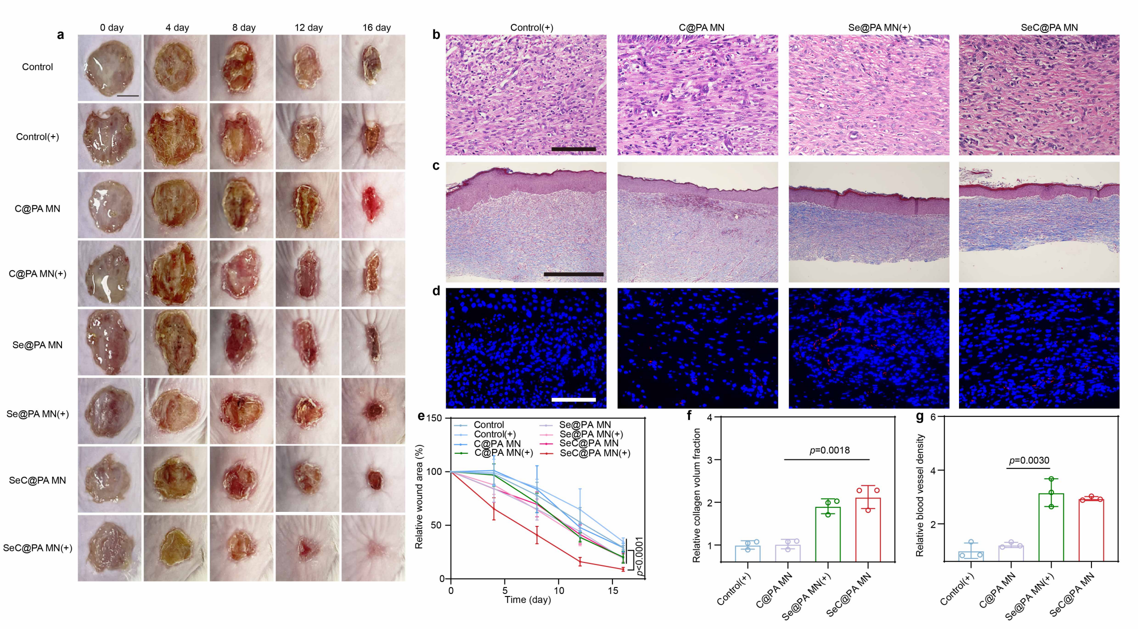

Supplementary Fig. 29. SeC@PA MN for promoting healing of biofilm-infected full-thickness diabetic wounds in rats. a,e Representative images of wounds during healing and quantitative data of relative wound area to day 16 for different groups at different time points (n = 6 biologically independent samples; mean ± SD). Scale bar is 5 mm. **b** Representative H&E staining images of wound samples treated with different groups on day 16. Scale bar is 100 µm**.** Three independent experiments were performed and representative results are shown. **c,f** Representative Masson's trichrome-stained images of wound samples and quantification of the collagen volume fraction for four groups on day 16 (n = 3 biologically independent samples; mean ± SD). Scale bar is 500 µm. **d,g** Representative immunofluorescence images of CD31 staining in the regenerated skin tissues and quantification of blood vessel density on day 16 after wound healing (n = 3 biologically independent samples; mean ± SD). Scale bar is 100 µm. Statistical significance was analyzed via one-way ANOVA with a Tukey post-hoc test. Source data are provided as a Source Data file. Control: blank microneedle; Control(+): blank microneedle under 660 nm irradiation (200 mW/cm^2) for 3 min; C@PA MN: microneedle containing Ce6-PDA-LA nanoparticles; C@PA MN(+): microneedle containing Ce6-PDA-LA nanoparticles under 660 nm irradiation (200 mW/cm^2) for 3 min; Se@PA MN: microneedle containing Se-PDA-LA nanoparticles; Se@PA MN(+): microneedle containing Se-PDA-LA nanoparticles under 660 nm irradiation (200 mW/cm^2) for 3 min; SeC@PA MN: microneedle containing Se-Ce6-PDA-LA nanoparticles; SeC@PA MN(+): microneedle containing Se-Ce6-PDA-LA nanoparticles under 660 nm irradiation (200 mW/cm^2) for 3 min.

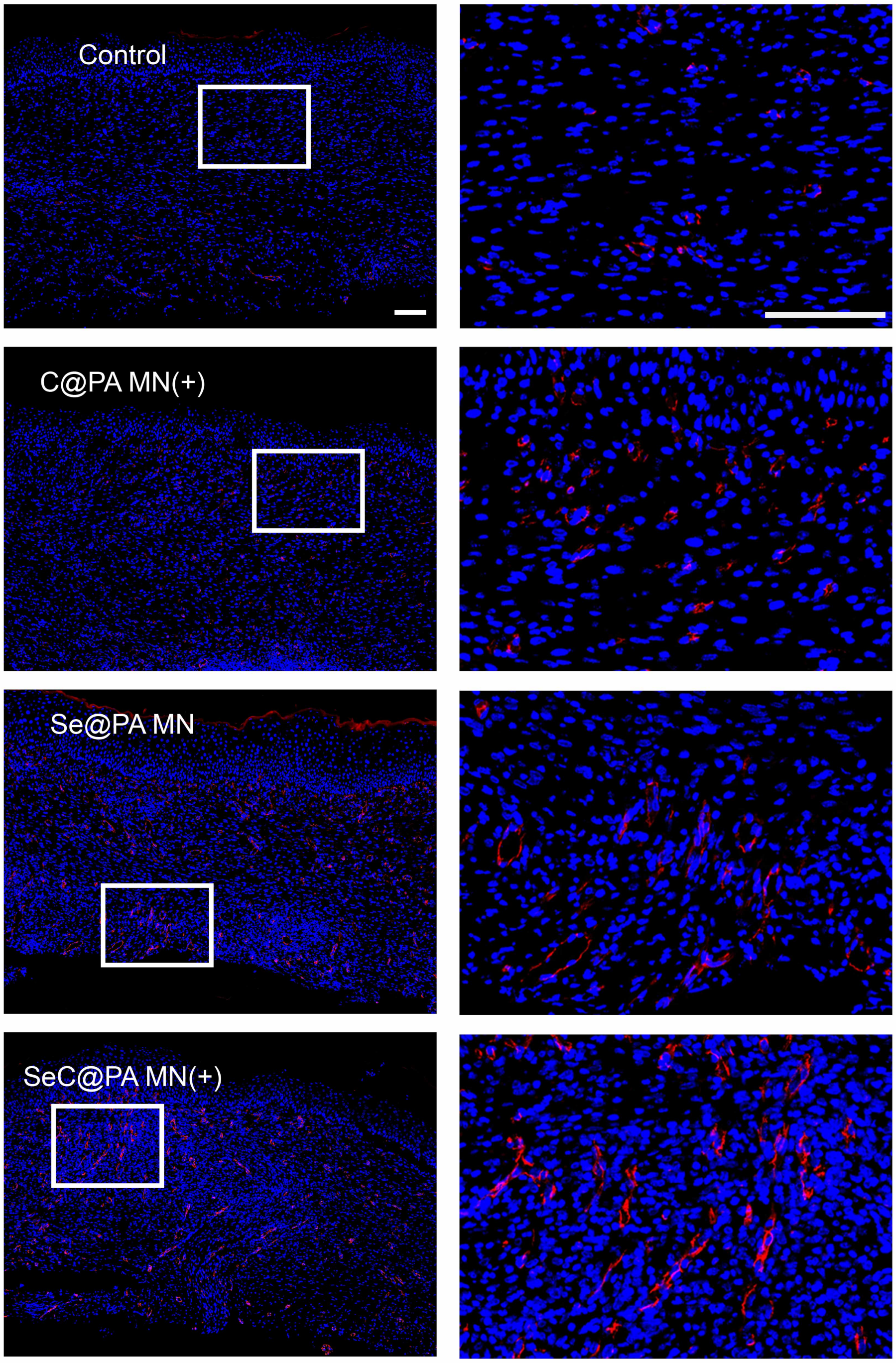

Supplementary Fig. 30. Representative immunofluorescence images of CD31 staining in the regenerated skin tissues on day 16 after wound healing. Scale bar is 100 μm. Control: blank microneedle; C@PA MN(+): microneedle containing Ce6-PDA-LA nanoparticles under 660 nm irradiation (200 mW/cm^2) for 3 min; Se@PA MN: microneedle containing Se-PDA-LA nanoparticles; SeC@PA MN(+): microneedle containing Se-Ce6-PDA-LA nanoparticles under 660 nm irradiation (200 mW/cm^2) for 3 min.

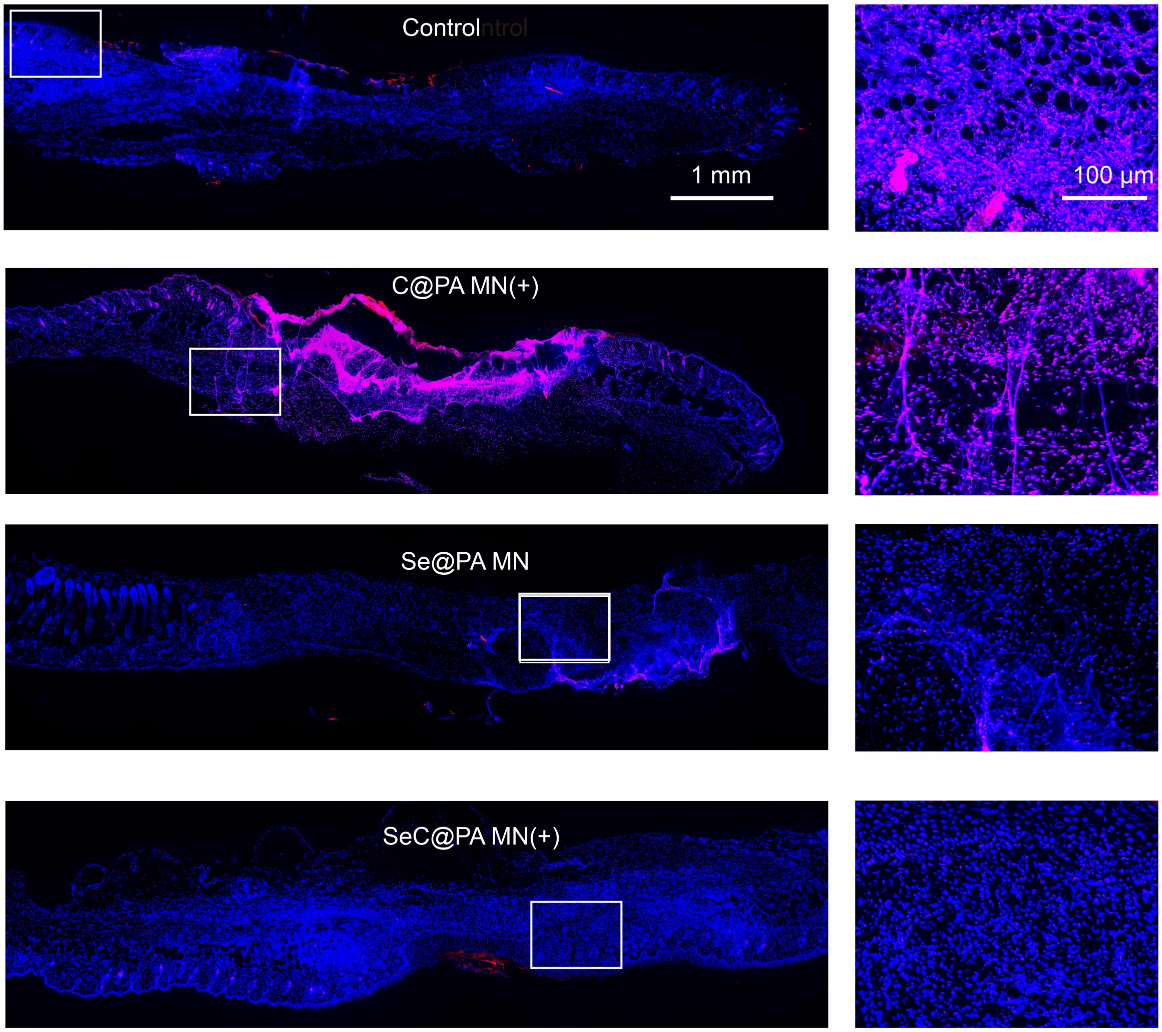

Supplementary Fig. 31. Representative immunofluorescence images of RS staining of different groups. Control: blank microneedle; C@PA MN(+): microneedle containing Ce6-PDA-LA nanoparticles under 660 nm irradiation (200 mW/cm^2) for 3 min; Se@PA MN: microneedle containing Se-PDA-LA nanoparticles; SeC@PA MN(+): microneedle containing Se-Ce6-PDA-LA nanoparticles under 660 nm irradiation (200 mW/cm^2) for 3 min.

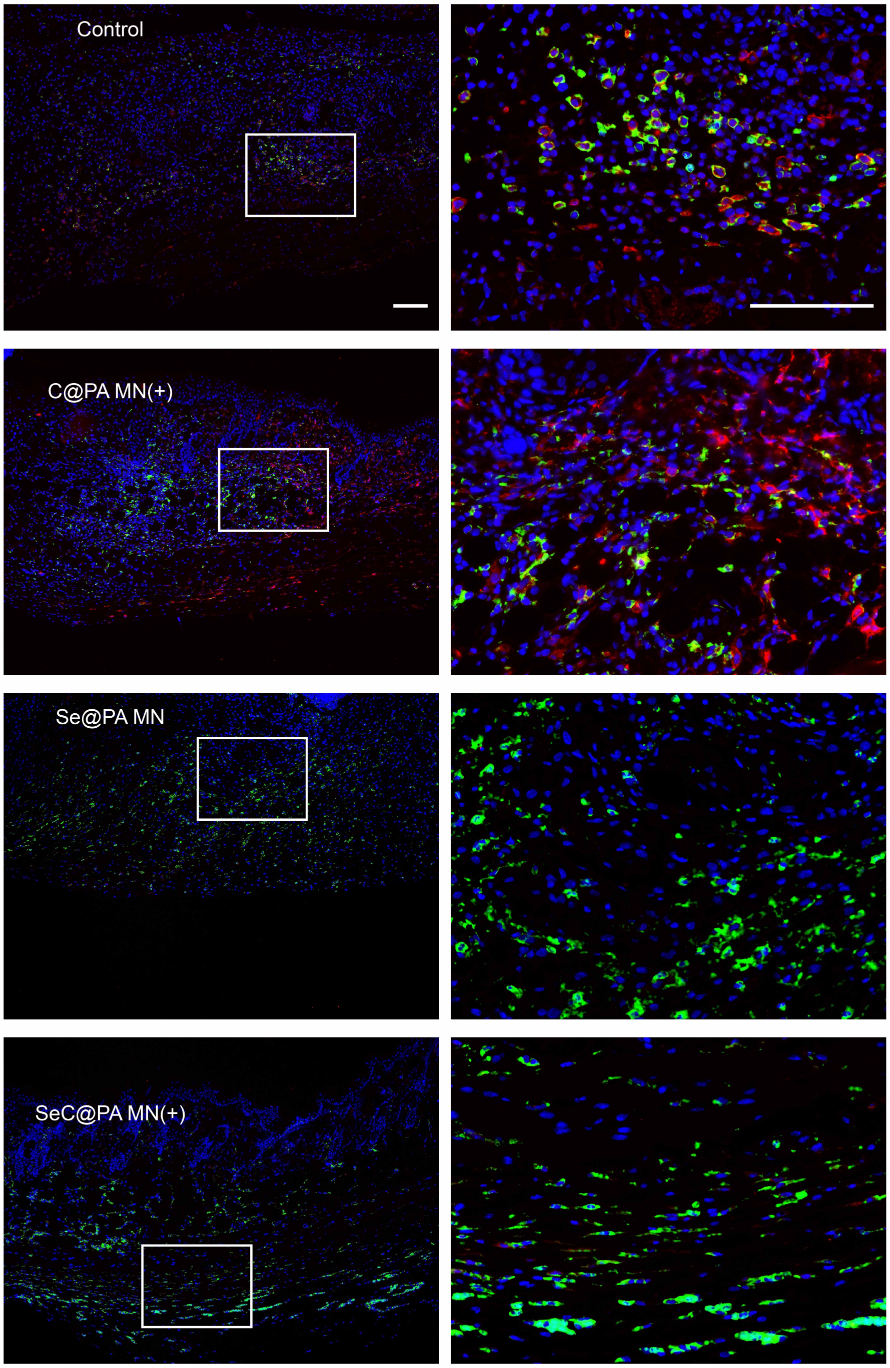

Supplementary Fig. 32. Immunofluorescence images of F4/80 (green) and CD86 (red) in the infected tissues at day 8. Scale bar is 100 μm. Control: blank microneedle; C@PA MN(+): microneedle containing Ce6-PDA-LA nanoparticles under 660 nm irradiation (200 mW/cm^2) for 3 min; Se@PA MN: microneedle containing Se-PDA-LA nanoparticles; SeC@PA MN(+): microneedle containing Se-Ce6-PDA-LA nanoparticles under 660 nm irradiation (200 mW/cm^2) for 3 min.

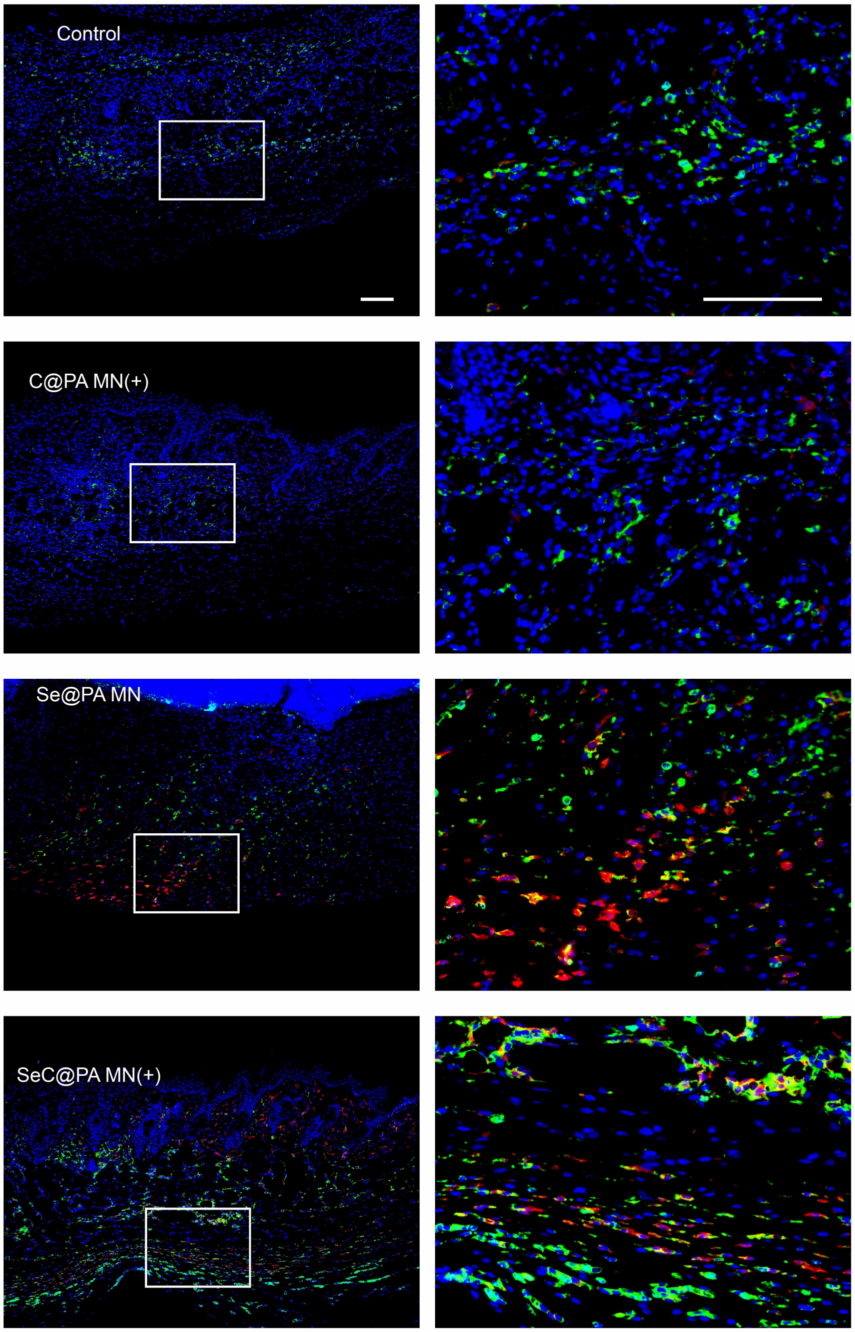

Supplementary Fig. 33. Immunofluorescence images of F4/80 (green) and CD206 (red) in the infected tissues at day 8. Scale bar is 100 μm. Control: blank microneedle; C@PA MN(+): microneedle containing Ce6-PDA-LA nanoparticles under 660 nm irradiation (200 mW/cm^2) for 3 min; Se@PA MN: microneedle containing Se-PDA-LA nanoparticles; SeC@PA MN(+): microneedle containing Se-Ce6-PDA-LA nanoparticles under 660 nm irradiation (200 mW/cm^2) for 3 min.

Supplementary Table 1. Loading efficiency of Se, Ce6 and LA in SeC@PA. Source data are provided as a Source Data file.

Se	Ce6	LA
23.23 ± 4.11 (%)	8.40 ± 4.38 (%)	12.60 ± 3.79 (%)

Supplementary Table 2. Primers used in the RT-PCR analysis.

Genes	Forward primer	Reverse primer
CD206	TGGCAAGTATCCACAGCA	GGTTCCATCACTCCACTCA
CD86	ACGGAGTCAATGAAGATTTCCT	GATTCGGCTTCTTGTGACATAC
IL-4ra	GCGTGCTTGCTGGTTCT	GTCCTGGGCTCCCTCTC
IL-10	GGAAGACAATAACTGCACCCACT	CAACCCAAGTAACCCTTAAAGTCC
Arg-1	CCCAGCTTGTCTACTTCAGTCATG	GGCAACCTGTGTCCTTTCTCCT
IL-1β	TGTGTTTTCCTCCTTGCCTCTGAT	TGCTGCCTAATGTCCCCTTGAAT
iNOS2	GCCCAGGAGGAGAGAGAT	GCAAAGAGGACTGTGGCT
TNF-α	CTTGTTGCCTCCTCTTTTGCTTA	CTTTATTTCTCTCAATGACCCGTAG
STAT6	TCTCCACGCTTCACATTG	GACCACCAAGGGCAGAGAC
GAPDH	GGAGCGAGATCCCTCCAAAAT	GGCTGTTGTCATACTTCTCATGG
